# Safety of red blood cell transfusion despite serologic incompatibility in autoimmune hemolytic anemia: A real-world multicenter study

**DOI:** 10.1007/s00277-026-07188-6

**Published:** 2026-07-23

**Authors:** Claudia Cammarata, Simona Raso, Marta Mattana, Riccardo Tomasello, Giulia Maria Camarda, Simona D’Angelo, Elisabetta Scirè, Maria Ilaria Dilena, Roberta Fedele, Sergio Siragusa, Mariasanta Napolitano

**Affiliations:** 1 PO. “Sant’Elia”, Caltanissetta, Italy; 2 Department of Health Promotion, Mother and Child Care, Internal Medicine and Medical Specialities (PROMISE), ITA University of Palermo, Palermo, Italy; 3 Department of precision medicine in medical, surgical and critical care (Me.Pre.Cc), University of Palermo, Palermo, Italy; 4 Hematology and Rare Diseases Unit, Azienda Ospedaliera Ospedali Riuniti Villa Sofia-Cervello, Palermo, Italy; 5 UOC Medicina Trasfusionale AOUP Paolo Giaccone, Palermo, Italy; 6 UOS Direzione Medica di Presidio - AOOR Villa Sofia- Cervello, Palermo, Italy; 7 UOC Medicina Trasfusionale e dei Trapianti, AOOR Villa Sofia- Cervello, Palermo, Italy

**Keywords:** Autoimmune Hemolytic Anemia, Transfusion, Red blood cells, Transfusion safety, Splenomegaly, Retrospective study

## Abstract

Red blood cell transfusion in Autoimmune Hemolytic Anemia (AIHA) poses significant clinical challenges due to pan-reactive autoantibodies that interfere with cross-matching, often raising concerns regarding the safety of transfusing serologically “incompatible” blood. This retrospective multicenter study evaluated the frequency, management criteria, and safety profile of red blood cell transfusions in a cohort of adult patients affected by AIHA with a specific focus on transfusion management in the setting of serologic incompatibility. We analyzed 93 adult patients diagnosed with AIHA at two hematology referral centers in Palermo, Italy, between 2020 and 2025. Transfusion support was required in 31.2% (*N* = 29) of patients. Decisions were mainly guided by a “double trigger” of low hemoglobin (mean 6.3 g/dL) and symptoms of tissue hypoxia, rather than by fixed hemoglobin thresholds. Notably, 61.1% of characterized units were serologically “incompatible” due to autoantibodies. Despite this, the adverse reaction rate was extremely low (1.2% per unit), with no hemolytic reactions recorded. This safety profile was likely supported by concomitant immunosuppressive therapy (89.6% of transfused patients), acting as “biological premedication”. Splenomegaly was identified as a marker for higher transfusion dependence (41.2% vs. 29.5%). Transfusion in AIHA is a safe and effective life-saving measure when managed with appropriate immunohematological oversight. Red blood cell transfusion in AIHA appears safe and effective when clinically indicated and managed with appropriate immunohematologic oversight, even in the presence of persistent serologic incompatibility. Transfusion support should not be delayed solely because of crossmatch incompatibility once clinically significant alloantibodies have been reasonably excluded.

## Introduction

Autoimmune Hemolytic Anemia (AIHA) comprises a heterogeneous group of rare hematological disorders characterized by the premature destruction of red blood cells (RBCs) mediated by autoantibodies directed against autologous erythrocyte antigens. While the prevalence of AIHA is relatively low, estimated in Western Europe at between 1 and 9 cases per 100,000 people, it represents a significant cause of acquired anemia that may evolve into severe, life-threatening clinical scenarios requiring rapid and multidisciplinary intervention [1, 2]. The clinical course is extremely variable, ranging from mild, self-limiting forms to rapidly progressive conditions with severe hemodynamic compromise.

The pathophysiology of AIHA is primarily classified based on the thermal characteristics of the autoantibodies involved. The most common form, Warm Autoimmune Hemolytic Anemia (wAIHA), accounts for approximately 70–80% of cases in adults [3]. It is mediated predominantly by polyclonal IgG antibodies that react optimally at body temperature (37 °C). These antibodies opsonize RBCs, leading to their sequestration and destruction by macrophages in the reticuloendothelial system, primarily within the spleen (extravascular hemolysis) [4]. In contrast, Cold Agglutinin Disease (CAD) involves IgM antibodies that bind to erythrocytes at lower temperatures (typically < 30 °C) in the peripheral circulation [5, 6]. The pentameric structure of IgM is highly efficient at fixing complement, leading to the deposition of C3b on the red cell membrane. This can result in extravascular hemolysis via hepatic clearance or, less frequently, intravascular hemolysis if the terminal complement complex is fully activated. Less common forms include mixed AIHA, characterized by the coexistence of warm and cold autoantibodies, and Paroxysmal Cold Hemoglobinuria (PCH), mediated by the biphasic Donath-Landsteiner IgG antibody [7, 8].

The management of AIHA centers on immunosuppressive therapies, such as corticosteroids, rituximab, and cytotoxic agents, aimed at reducing antibody production. However, in the acute setting of severe anemia with symptomatic hypoxia, red blood cell transfusion often becomes a necessary, albeit temporary, life-saving measure. Transfusion in AIHA is historically considered a high-risk procedure due to complex immunohematological challenges [9, 10]. The presence of autoantibodies, which are often “pan-reactive” against common RBC antigens, frequently results in a positive cross-match for all available donor units. This phenomenon complicates the detection of underlying alloantibodies, i.e. antibodies against non-self-antigens, developed from prior pregnancies or transfusions, which may be masked by the autoantibody [11].

The inability to find serologically compatible blood creates a significant clinical dilemma. Physicians must balance the immediate risk of hypoxic death due to severe anemia against the potential risk of a hemolytic transfusion reaction. In clinical practice, transfusion medicine services often select units showing the lowest degree of serologic reactivity when completely compatible blood is unavailable. However, the clinical significance of the strength of in vitro incompatibility remains controversial [12–14]. Moreover, the concept of transfusing incompatible blood remains counterintuitive and a source of anxiety for many clinicians. Furthermore, there is a lack of universally accepted guidelines defining precise hemoglobin (Hb) thresholds or clinical triggers for transfusion in this specific population. The decision is often left to individual clinical judgment, leading to variability in practice regarding the use of “least incompatible” units and the administration of premedication [8].

Recent studies have also highlighted the association between AIHA and thrombotic events, further complicating management [15]. The release of free hemoglobin and microparticles during hemolysis can induce a prothrombotic state, necessitating a careful evaluation of the patient’s overall hemodynamic and coagulation status [16]. Despite these complexities, transfusion remains a critical pillar of support.

The primary objective of this study was to contribute to the body of knowledge regarding transfusion practices in AIHA through a retrospective multicenter analysis. Specifically, this study aimed to determine the frequency and modalities of RBC transfusions in a real-world cohort, evaluate the clinical and laboratory parameters guiding the decision to transfuse (e.g., Hb levels vs. symptoms), and assess the safety of “least incompatible” transfusions (showing the weakest serologic reactivity with the patient's blood) in terms of adverse reactions and alloimmunization. By analyzing “real-life” data from two hematology referral centers, this research seeks to provide empirical evidence to support more standardized and safe transfusion strategies for patients with AIHA.

## Materials and methods

This retrospective, multicenter observational study analyzed a cohort of adult patients diagnosed with AIHA. The study was conducted at two major hematology referral centers in Palermo, Italy: the Hematology Unit of the “Paolo Giaccone” University Hospital and the Hematology and Rare Disease Unit of “Villa Sofia-Cervello” Hospital. The observation period spanned five years, from September 2020 to September 2025. The study protocol was approved by the Institutional Review Boards (IRBs) of both participating centers. Written informed consent for publication was obtained from the patients.

The cohort comprised 93 patients with a confirmed clinical and laboratory diagnosis of AIHA. Inclusion criteria encompassed all AIHA subtypes (warm, cold, mixed, and atypical) and both primary and secondary etiologies, irrespective of transfusion requirements during the study period. Diagnosis was defined by the concomitant presence of anemia, a positive Direct Antiglobulin Test (DAT), and altered hemolysis markers, such as elevated lactate dehydrogenase (LDH) and/or indirect bilirubin, in accordance with international diagnostic criteria [2]. No standardized transfusion protocol was in place across the participating centers. Transfusion decisions were made by the treating physician in collaboration with the transfusion medicine service, which generally selected ABO- and Rh-compatible units after exclusion, whenever feasible, clinically significant alloantibodies. To ensure cohort homogeneity, exclusion criteria included pediatric patients, pregnant women, and recipients of allogeneic hematopoietic stem cell transplantation (HSCT). Furthermore, cases of non-autoimmune hemolytic anemia (e.g. hemoglobinopathies, microangiopathic hemolysis, drug-induced non-immune hemolysis) and patients with insufficient clinical or laboratory data for accurate retrospective assessment were excluded. Data were retrospectively retrieved from medical records and clinical registries. Baseline variables included demographics (age at diagnosis, gender), AIHA subtype and etiology, and the diagnostic setting. Key laboratory parameters recorded included baseline Hb levels, hemolysis markers (LDH), DAT intensity scores, and cold agglutinin titers. Consistent with the study’s primary objective, the analysis focused heavily on transfusion management, when available. Transfusion-related variables included transfusion rates, pre-transfusion hemoglobin levels, and the clinical symptoms prompting transfusion (e.g., angina, dyspnea, asthenia). Transfusion burden and efficacy were evaluated based on the number of units administered and post-transfusion Hb increments. Immunohematological data included the serological compatibility of transfused units (categorized as “compatible” or “incompatible”) and the incidence of adverse transfusion reactions. All transfusions were ABO- and RhD-compatible; incompatibility refers exclusively to autoantibody-mediated crossmatch reactivity. When feasible, alloantibodies were excluded using available serological methods; however, in emergency settings, extensive adsorption studies could not always be performed before transfusion. Additionally, splenomegaly was recorded to investigate potential correlations with transfusion requirements and disease severity. The number of therapeutic lines was used as a proxy for disease refractoriness.

Data processing and statistical analysis were performed using the open-source software R. Descriptive statistics included means, medians, standard deviations, and ranges for quantitative variables (e.g., Hb, LDH, age), and frequencies/percentages for categorical variables (e.g., gender, AIHA subtype). Comparative analyses assessed differences between subgroups, specifically between transfused and non-transfused patients, and between those with and without splenomegaly. Associations between variables, such as the correlation between splenomegaly and therapeutic lines, were evaluated using appropriate statistical tests (e.g., Fisher’s Exact Test), acknowledging the limitations imposed by sample size and retrospective design.

## Results

The study cohort comprised 93 patients with a confirmed diagnosis of AIHA. The median age at diagnosis was 66 years (Interquartile Range [IQR]: 57.3–75.8), with a predominance of females (63.4%, *N* = 59) (Table 1).

**Table 1:** Demographics Data and Baseline laboratory parameters

Study population, n	93
Median age, years (IQR)	**66** (58–76)
Gender, n (%)	**F 59** (63%) - **M 34** (37%)
Concomitant medications, n (%)	**26/29 (89**.**6%)**
Hb, g/dL (range)	**7**.**7 L (2**.**8–10**.**3)**
Diagnostic setting, n (%)	**Outpatient (51%) - ER (36%)**
DAT	**+++ (54**.**3%); ++++ (45**,**7%)**
Splenomegaly, n (%)	**34 (43**.**6%)**

*Abbreviations: Hb* Hemoglobin, *DAT* Direct Antiglobulin Test

Immunohematological classification was successfully determined for 87.1% of the cohort (*N* = 81). Consistent with global prevalence data, Warm-AIHA (wAIHA) was the predominant subtype, identified in 66.7% of classified patients (*N* = 54). Cold forms (cAIHA/CAD) accounted for 28.4% (*N* = 23), while mixed forms were rare, affecting only 4.9% of patients (*N* = 4). Among patients with wAIHA, the Direct Antiglobulin Test (DAT) was strongly positive in all evaluable cases (+++ in 54.3% and ++++ in 45.7%) (Table 2). The clinical presentation at diagnosis varied significantly in severity. The mean Hb level was 7.7 g/dL, but the range was extremely wide (2.8–10.3 g/dL), indicating that while some patients presented with mild anemia, others presented with life-threatening anemia. Regarding the setting of diagnosis, 51.1% of patients were diagnosed in an outpatient setting, while a significant proportion (35.9%) presented to the Emergency Department, suggesting an acute and symptomatic onset for more than one-third of the population.

**Table 2:** Immunohematologic classification

wAIHA n (%)	54 (66.7%)
Idiopathic CAD, n (%)	**14 (60**.**9%)**
Secondary CAD, n (%)	**9 (39**.**1%)**
Mixed AHIA, n (%)	**4 (4**.**9%)**
g/dL	**8**

*Abbreviations: wAHIA,* warm Autoimmune Hemolytic Anemia, *CAD* Cold Agglutinin Disease

Transfusion support was required in 31.2% of the cohort (29 out of 93 patients), while the remaining 68.8% were managed with medical therapy alone. The decision to administer RBC transfusions was not based on a single hemoglobin threshold. The mean pre-transfusion Hb was 6.3 g/dL (range 2.8–8.7 g/dL). However, the decision was strongly correlated with clinical signs of decompensation. Transfusions were administered to patients exhibiting severe asthenia (*N* = 12), angina pectoris (*N* = 3), altered mental status/stupor (*N* = 1), acute respiratory failure (*N* = 1), and in patients with a history of myocardial infarction (*N* = 1). This pattern supports a “double-trigger” strategy involving both laboratory values and clinical symptoms (Table 3).The transfusion burden was heterogeneous. The majority of transfused patients (55.2%, *N* = 16) received a single unit in an emergency setting. A smaller subgroup (31.0%, *N* = 9) required 2–3 units, while 13.8% (*N* = 4) demonstrated significant transfusion dependence, requiring more than 3 units (up to 14 units in one case). The median transfusion load was 2 units. Transfusion served as an effective bridge therapy, raising the mean Hb from 6.3 g/dL to a post-transfusion value of 8.8 g/dL.

**Table 3:** Clinical and Transfusion Characteristics of Transfused Patients

Transfused patients	29 (31.2%)
Hb pre-Tx, g/dL (range)	**6**.**3 (2**.**8–8**.**7)**
Hb post-Tx, g/dL (range)	**8** **8**
Transfusion burden (range)	
On therapy, n (%)	**26 (89**.**6%)**
Transfusion reactions, n	**1/84**
Least incompatible Units, n (%)	**11/18 (61**.**1%)**

*Abbreviations: TX*: transfusion

A critical aspect of this study was the assessment of transfusion safety in the setting of serological incompatibility. Compatibility data were retrieved for 18 transfused units. Of these, 11 units (61.1%) were administered despite a serologically “incompatible” cross-match, reflecting the inherent challenge of securing compatible blood in the presence of pan-reactive autoantibodies. Specifically, these units demonstrated incompatibility in both the polyspecific anti-human globulin (AHG) phase, and in reverse grouping, exhibiting reactivity across both warm and cold thermal amplitudes. Due to the clinical urgency, extensive adsorption studies could not be performed prior to transfusion to definitively exclude underlying alloantibodies.

Despite the high rate of incompatibility, the safety profile was excellent. Across a total of 84 units administered to 29 patients, only one adverse reaction was recorded (1.2% per unit). The reaction was characterized by chills and angina, was non-hemolytic, and completely resolved with hydrocortisone administration. Importantly, none of the 11 transfusions performed despite persistent serologic incompatibility resulted in a hemolytic reaction. It is noteworthy that at the time of transfusion, 89.6% (26/29) of patients were actively receiving immunosuppressive therapy (steroids as single agent or combined with Rituximab or IVIG).

Spleen size was assessed by abdominal ultrasound evaluation in 78 patients, and splenomegaly was identified in 43.6% (*N* = 34) of patients. The median spleen length in this group was 15 cm (11–23). We analyzed whether splenomegaly could help define disease severity. Surprisingly, patients with splenomegaly did not present with lower hemoglobin levels or higher LDH at diagnosis compared to those without splenomegaly (Hb 7.81 vs. 7.55 g/dL; LDH 562.3 vs. 653.6 U/L) - (*p* = 0.6219; *p* = 0.3496). However, there was a notable difference in transfusion requirements. Patients with splenomegaly were more likely to be transfused (41.2%) compared to those without splenomegaly (29.5%), although this difference showed a trend toward significance (*p* = 0.0897), likely limited by sample size (Table 4). Additionally, when analyzing therapeutic refractoriness, 76.0% of patients with splenomegaly required two or more lines of therapy, compared to 72.0% of those without, suggesting a trend toward a more difficult-to-treat phenotype in patients with splenic enlargement.

**Table 4:** Association between splenomegaly and clinical parameters

Analysis	*P*- Value
Spleen size vs. Hb	0,6219
Spleen size vs. LDH	0,3496
Spleen size vs. transfusion requirement	0,0897

* Abbreviations: Hb* Hemoglobin, *LDH* lactate dehydrogenase, *TX* transfusion

## Discussion and conclusions

This multicenter retrospective study provides a comprehensive analysis of the real-world management of AIHA from two reference centers, with a specific focus on the efficacy and safety of transfusion support. Our findings offer significant insights that challenge historical hesitancies regarding transfusion in this patient population and support a more personalized clinical approach.

The most impactful finding of our analysis lies in the confirmation of the safety of transfusing serologically “incompatible” RBC units. In our cohort, over 60% of characterized units were incompatible in vitro due to pan-reactive autoantibodies. However, no hemolytic reactions occurred and the overall reaction rate was only 1.2%. Our findings support the concept that clinically indicated transfusion in AIHA can be safely performed despite persistent serologic incompatibility, provided that clinically significant alloantibodies (e.g., anti-Rh, anti-Kell) are reasonably excluded and transfusion support is managed collaboratively with transfusion medicine specialists [17–19], as recommended by current transfusion guidelines. The fear of “fueling the fire” of hemolysis often leads to delays in life-saving support; our data suggest that such delays are unwarranted when proper protocols are followed. Transfusion should thus not be delayed in critically anemic AIHA patients solely because of persistent serologic incompatibility [20].

We observed that nearly 90% of transfused patients were receiving concurrent immunosuppressive therapy, primarily high-dose corticosteroids, at the time of transfusion. We hypothesize that concomitant immunosuppressive therapy may act as a form of ‘biological premedication’. Corticosteroids are known to suppress the phagocytic activity of macrophages in the reticuloendothelial system, which is the primary site of extravascular hemolysis in wAIHA [21]. By initiating therapy prior to or during transfusion, clinicians may effectively blunt the immune system’s capacity to destroy the transfused donor RBCs. This “dampening” effect may contribute to the remarkably low reaction rate observed in our study (1.2%) compared with other cohorts reporting reaction rates up to 7% [22]. This finding supports the recommendation to always pair transfusion with aggressive immunosuppression.

Our analysis of decision-making criteria highlights the absence of a rigid hemoglobin threshold for transfusion in real-world practice. While the mean pre-transfusion Hb was 6.3 g/dL, the decision was invariably linked to symptoms of tissue hypoxia (angina, asthenia, altered mental status). This aligns with the “double trigger” approach (clinical + laboratory) endorsed in critical care and anemia management guidelines [23, 24]. In AIHA, where the onset of anemia can be rapid, physiological compensation mechanisms (or lack thereof) are more critical determinants of transfusion need than the absolute Hb number. The effectiveness of this approach is supported by the successful stabilization of patients and the mean post-transfusion Hb increment to 8.8 g/dL.

The analysis of splenomegaly revealed an interesting clinical paradox. Enlarged spleens did not correlate with lower Hb at diagnosis, yet they predicted a higher likelihood of requiring transfusion (41.2% vs. 29.5%). We suggest that splenomegaly in AIHA may serve as a marker of a chronic, hyper-hemolytic phenotype that is “compensated” (maintaining similar baseline Hb) but fragile. These patients may have a larger mass of reticuloendothelial tissue actively sequestering RBCs, making them more prone to decompensation and less responsive to initial steroid monotherapy, thereby requiring transfusion support and additional lines of therapy. While statistical significance was limited by sample size, the trend suggests that spleen size should be considered in risk stratification for transfusion dependence.

This study has several limitations related to its retrospective structure. Availability and completeness of transfusion-related immunohematologic data were variable, and compatibility assays were not documented for all units, which may have led to an underestimation of serological findings or alloimmunization events. Our cohort is relatively large for a rare disease such as AIHA; however the sample size of specific subgroups (i.e., patients with splenomegaly or those receiving multiple transfusions) is limited, thus impairing the ability to draw firm causal inferences. The absence of a standardized transfusion protocol across centers may also have influenced both transfusion thresholds and adverse event reporting. Additionally, because patients were receiving concomitant immunosuppressive therapies at the time of transfusion, it is difficult to isolate the independent effect of “least incompatible” transfusions on safety outcomes, but this mirrors common clinical practice. These limitations imply that the current findings are “hypothesis-generating” and highlight the need for prospective, standardized studies to better define transfusion strategies in AIHA. These findings reinforce the importance of collaboration between hematologists and transfusion medicine specialists in AIHA management.

In conclusion, this study demonstrates that RBC transfusion in AIHA acts as a safe and effective life-saving measure. In our cohort, red blood cell transfusion in AIHA was safe and clinically effective even in the presence of serologic incompatibility, when supported by appropriate immunohematologic evaluation and clinical monitoring. Clinical urgency and symptoms of tissue hypoxia should remain central in transfusion decision-making. Consequently, clinical management should move away from fixed thresholds in favor of a “double trigger” model in AIHA guided by hypoxia symptoms and hemoglobin trends. The concomitant immunosuppression may have contributed to the favorable safety profile observed in our cohort. Finally, splenomegaly emerged as a potentially relevant prognostic marker, identifying a specific subgroup of patients characterized by higher transfusion requirements and a potentially more refractory clinical course.

## Data Availability

The datasets generated during and/or analyzed during the current study are available from the corresponding author on reasonable request.
